# Statin Short-term Inhibition of Insulin Sensitivity and Secretion During Acute Phase of ST-Elevation Myocardial Infarction

**DOI:** 10.1038/s41598-019-52111-x

**Published:** 2019-11-08

**Authors:** Andrei C. Sposito, Luiz Sergio F. Carvalho, Filipe A. Moura, Alessandra M. Campos-Staffico, Riobaldo M. R. Cintra, Wilson Nadruz, Osorio R. Almeida, Jose C. Quinaglia e Silva

**Affiliations:** 1 Faculty of Medical Sciences, State University of Campinas (Unicamp), Campinas, SP Brazil; 2 Department of Medicine, Weill-Cornell Medical College, New York, New York, United States; 3 Hospital de Base do Distrito Federal, Brasília, Brazil

**Keywords:** Carbohydrates, Myocardial infarction

## Abstract

Hyperglycemia during myocardial infarction (MI) has a strong and direct association with mortality. In stable patients and experimental models, statins favor the elevation of glycaemia. The present study investigated whether short-course treatment with statins during MI can influence glucose homeostasis and thus the clinical outcome. In this prospective study, euglycemic hyperinsulinemic clamp (EHC) was performed at second (D2) and sixth (D6) day after MI in patients randomized to simvastatin (S)10 or 80 mg/day during hospitalization (n = 27). In addition, patients (n = 550) were treated without (WS) or with simvastatin (S) at 20, 40 or 80 mg/day had HOMA2S on admission (D1) and fifth (D5) day after MI. According to EHC, insulin sensitivity increased by 20 ± 60% in S10 and decreased by −6 ± 28% in S80 (p = 0.025). Consistently, the changes in HOMA2S between D1 and D5 were 40 ± 145% (WS), 22 ± 117% (S20), 16 ± 61% (S40) and −2% ± 88% (S80) (p = 0.001). In conclusion, statin during the acute phase of MI reduces insulin sensitivity in a dose-dependent manner.

## Introduction

Both hyperglycemia and hypoglycemia during the acute phase of myocardial infarction (MI), particularly in those with ST-elevation MI (STEMI)^1,2^, has evoked clinical concern ever since it was established as a robust predictor of worse short- and long-term prognosis^3,4^. From a mechanistic standpoint, hyperglycaemia can reduce thrombolysis, increase platelet aggregation, induce coronary vasoconstriction, decrease oxygen transport, and prolong endothelial inflammation after MI^4–6^. Consistently, observational data indicate that glucose normalization is associated with improved survival in hyperglycaemic patients hospitalized with MI^7^. In fact, in the only randomized controlled trial with MI patients where blood glucose was successfully reduced, glucose-lowering therapy improved clinical outcome^8^.

Regardless of acute phase glycaemia, the residual risk of death in patients with MI remains high. Mitigation of this residual risk has been attempted through the inclusion of high dose of potent statins in the early treatment of acute coronary syndromes (ACS), a rationale that was largely based on a broad range of potentially beneficial mechanisms^9^, even though evidence from clinical trials does not fully support this practice^10,11^. Indeed, we recently reported the lack of benefit with high doses of statin in 4,191 patients in the first 30 days after ACS^12^.

A factor that may possibly contribute for the lack of benefit has been raised in studies demonstrating increased insulin resistance (IR) after chronic statin therapy^11,13^. In line with this, in cellular and animal models, a decrease in both insulin sensitivity and secretion can occur immediately after statin administration^14,15^. Consequently, it can be hypothesized that statin treatment initiated in the acute phase of MI may favor hyperglycaemia during the acute phase. If this is so, such negative effect may indeed restrict or even surpass a spectrum of beneficial mechanisms related to the use of these drugs during ACS^9^. To date, this hypothesis has not been investigated. Hence, this study aimed to identify the influence of statins on glucose homeostasis during MI.

In the present study, we selected non-diabetic patients to obtain a sample of individuals whose metabolic state reflects a direct and self-adjusted relationship between sensitivity and insulin secretion. We also chose individuals admitted with STEMI because they traditionally have large myocardial lesions and are therefore more susceptible to changes in glucose homeostasis due to the potential neurohumoral and inflammatory stimuli^1^. We used two methods for measuring insulin sensitivity whose consistency was validated by our group in STEMI patients^16^. Euglycemic hyperinsulinemic clamps (EHC) were used for being the gold standard method in a small group of patients and the Homeostasis Model Assessment (HOMA) was used for being a fast and accessible index feasible to be applied in a larger group of STEMI patients allowing us to confirm the findings and investigate interactions.

## Materials and Methods

### Study designs

We prospectively enrolled STEMI patients referred to Hospital de Base do Distrito Federal, Brasilia, Brazil, who survived hospital course and were able to undergo glucose homeostasis assessments as described below. Inclusion criteria for both substudies were as follows: (i) less than 24 hours after the onset of MI symptoms, (ii) ST-segment elevation of a least 1 mm (frontal plane) or 2 mm (horizontal plane) in two contiguous leads, and (iii) myocardial necrosis, as evidenced by increase to at least one value above the 99^th^ percentile above the reference limit of CK-MB (25 U/L) and troponin I (0.04 ng/mL) followed by a decline of both. Exclusion criteria were glycosylated hemoglobin (HbA1c) ≥ 6.5%, prior diagnosis of diabetes or use of antidiabetic therapies. The Institutional Ethics Committee, Comitê de Ética em Pesquisa da Secretaria de Estado de Saúde do Distrito Federal (CEP/SES-DF), approved protocols of both substudies. All methods were performed in accordance with the relevant guidelines and regulations.

In the substudy 1 (interventional), consecutive STEMI patients (n = 37) aged between 20 and 60 years were prospectively enrolled into this substudy to investigate the effect of statin therapy on insulin sensitivity (ClinicalTrials.gov: NCT01205347). Exclusion criterium was the use of statins in the last 6 months. After enrollment, 10 patients were excluded for not completing the second assessment. The reasons for exclusion were withdrawal of consent (n = 4) and significant deterioration of clinical status (n = 6), i.e. death, cardiogenic shock, or recurrent coronary event. Patients were randomized at the second day of hospitalization (D2) for treatment with simvastatin (Zocor^®^, Merck, Sharp & Dohme, São Paulo, Brazil) 10 mg/day (n = 13) or 80 mg/day (n = 14). Although there is concern about the risk of myopathy with simvastatin 80 mg/day, the decision to use this dose was based on the wide availability of this statin in the public health system where enrolled patients were followed up over the period in which this substudy was carried out.

The EHC were performed after a 12-h fast at D2 and at the sixth day (D6) of STEMI onset. EHCs were not done at the first day of hospitalization (D1) in order to avoid delaying of medical assistance in the first 24 hours of STEMI and to ensure a 12-h overnight fasting period. As EHC is a laborious and time-consuming procedure, a limited number of patients was enrolled in substudy 1. Accuracy validation of the substitution indices for the insulin sensitivity (IS) obtained by the EHC in MI patients was performed by our group and published elsewhere^16^. MI mass was measured by cardiac magnetic resonance imaging (CMRi) 30 days after STEMI to verify whether there was imbalance of infarcted mass between groups. Blood samples were collected at admission (D1) and at the fifth day after STEMI onset (D5) for biochemical analyses.

Substudy 2 (observational) was designed to evaluate, in a multivariate approach, the interaction between statin use and IR induction. Hence, 375 consecutive participants of the prospective observational cohort *Brasilia Heart Study* (ClinicalTrials.gov: NCT02062554, retrospectively registered) were enrolled. A complete medical evaluation was performed upon hospital admission (D1) followed by an initial blood sample collection with a mean fasting time of 8 ± 3 hours. The second sample was collected after a 12-h overnight fast at fifth day of hospitalization (D5). Attending physicians exclusively decided on medical treatments without any participation of the investigators and were blinded to all results from the methods used in this study. At the end of data collection patients were grouped according to the use of statin during hospitalization: without statin (WS), simvastatin 20 mg/day (S20), simvastatin 40 mg/day (S40) and simvastatin 80 mg/day (S80).

**Figure 1:**
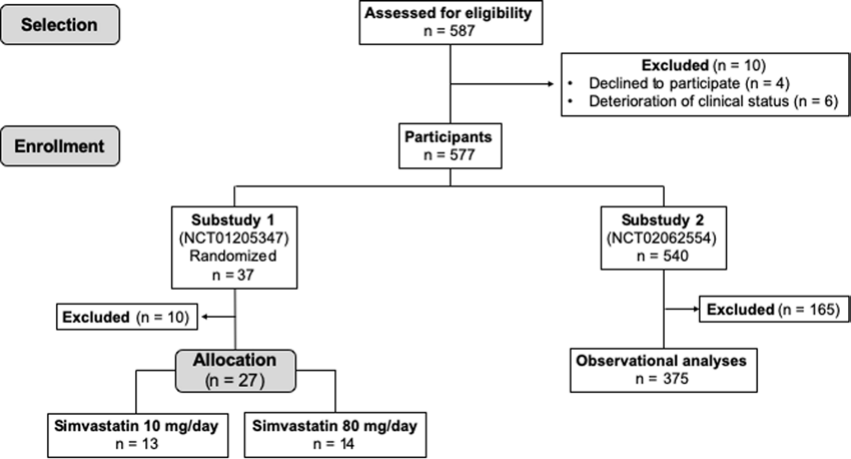
Diagram flow of substudies 1 and 2.

Both substudies were developed in compliance with STROBE and CONSORT guidelines. Diagram Flow of substudies can be found in the Fig. 1.

### Laboratory analyses

The following plasma measurements were performed: glucose (Glucose GOD-PAP, Roche Diagnostics, Mannheim, Germany, coefficient of variation 1.7%, range 241 mg/dL), total cholesterol (CHOD-PAP, Roche Diagnostics, Mannheim, Germany, coefficient of variation 4.0%, range 318 mg/dL), triglycerides (GPO-PAP, Roche Diagnostics, Mannheim, Germany, coefficient of variation 6.4%, range 117 mg/dL), high-density lipoprotein cholesterol (HDL-C) (HDL-C without sample pre-treatment, Roche Diagnostics, Mannheim, Germany, coefficient of variation 4.1%, range 74 mg/dL), HbA1c (Variant II, Bio-Rad Laboratories, Hercules, CA, USA), high-sensitivity C-reactive protein (CRP) (Cardiophase, Dade Behring, Marburg, Germany, coefficient of variation 6.7%, 13.8 g/L). Low-density lipoprotein cholesterol (LDL-C, range 280 mg/dL) was calculated by the Friedewald formula. Technicians, who were blinded to the statin treatment, performed all laboratorial analyses.

### Glucose homeostasis model assessment

We employed the HOMA Calculator version 2.2.2 in order to assess insulin sensitivity (HOMA2S), based on plasma insulin, and β cell function (HOMA2B), based on plasma C-peptide, in all enrolled patientes^17^. Insulin (Roche Diagnostics, Mannheim, USA, coefficient of variation 4.2%, range 117 µI/mL) and C-peptide (Immulite 2000, Diagnostic Products Corporation, Los Angeles, CA, USA, coefficient of variation 7.1%, range 22.6 ng/dL) were measured on first day (D1) and fifth day of admission (D5). The Disposition Index was calculated as HOMA2B*HOMA2S/100.

### Euglycemic hyperinsulinemic clamps

EHCs were performed at 07:00-h on second day (D2) and sixth day (D6). In order to arterialize and collect blood samples, we heated the arm to 50 °C, catheterized the antecubital vein and maintained its patency with slow saline drip. In the contralateral arm, we performed insulin and glucose infusion and 30 minutes after the beginning of the infusion, we started collecting glucose and insulin plasma concentrations. During 180 minutes, we infused insulin (Novolin R®; Novo-Nordisk, Bagsvaerd, Denmark) at a rate of 7 pmol.kg^–1^.min^–1^ and maintained euglycemia (~100 mg/dL) by simultaneous 50% glucose intravenous administration whose infusion rate was corrected every 10 minutes. The insulin sensitivity index (ISi) was calculated in two steps by using the equation ISi = M/(G × ΔI) corrected for body weight. M is the steady state glucose infusion rate (milligrams per min), G is the steady-state blood glucose concentration (mg/dL), and ΔI is the difference between basal and steady state plasma insulin concentrations (micro units/mL). ISi was estimated by increasing fractional glucose disappearance for each unit increase in plasma insulin^18,19^.

### Cardiac magnetic resonance imaging (CMRI)

CMRI studies were carried out in the intervention group using a MRI scanner with a 1.5-T (Signa CV/i, GE Medical Systems, Waukesha, WI), equipped with a gradient of high performance (gradient strength 40mT/m; maximum slew rate 150 mT/m/s) and a four-elements phased array cardiac coil. MI mass was quantified using gadolinium-based delayed enhancement myocardial images thirty days after STEMI.

### Statistical methods

Data were presented as mean ± SD for normally distributed data or median and interquartile range (IQR) for skewed data. Comparisons between S10 and S80 groups in the interventional substudy were made by Wilcoxon-Mann-Whitney test or a two-tailed *t* test. Baseline patient characteristics were stratified by the dose of simvastatin given using ANOVA 1-way test or Fisher exact test when appropriate. Categorical variables were compared by using the chi-square test. Analysis of covariance (ANCOVA) was used to assess the association between the treatment arm and the change in glycaemia, insulin, C-peptide, HOMA2S, and HOMA2B from D1 to D5, all of which were calculated as a delta, i.e. D5-D1. Adjustments for baseline levels, age, and gender were performed in all comparisons between simvastatin groups. Assumptions of the ANCOVA models (linearity, normality of distribution and equal variance) were checked using histograms, normal probability plots, and residual scatter plots.

Principal components factor analysis (PCA) was performed retaining factors with eigenvalues of at least 1.0. Missing variables were handled by listwise deletion, i.e. including only those sets of data where all the variables are present. PCA is a method of identifying patterns in data and only is appropriate for use with highly correlated variables. This statistical approach groups linear combinations into principal components of correlated variables, but the components themselves are derived so that they are uncorrelated with each other. Variables with an absolute value of the coefficient ≥0.5 were considered to be influential within each factor. Variables included in the analysis were Delta HOMA2S, Delta HOMA2B, and Delta Disposition Index. A two-sided p-value of 0.05 was considered statistically significant. All analyses were performed with SPSS 20 and Stata 13 for Mac. The authors had full access to the data and take responsibility for its integrity. All authors have read and agree to the manuscript as written.

## Results

### Interventional substudy

#### Baseline characteristics

There were no significant differences between simvastatin groups regarding sex, gender, clinical characteristics upon admission, or anthropometric characteristics. Infarction mass, reperfusion therapy, and the use of beta-blockers were also similar (Table 1). Admission levels of glycaemia, LDL-C, HDL-C, triglycerides, and CRP were not different.

**Table 1:** Baseline characteristics and insulin sensitivity index (ISi) of patients selected to the interventional phase.

	S 10 mg	S 80 mg	*p*-value
N	13	14	
Age, years	54 ± 5	58 ± 9	0.8
Male gender, %	80	70	0.8
Heart rate, bpm	69 ± 11	70 ± 12	0.8
Systolic BP, mmHg	144 ± 24	143 ± 31	0.7
Diastolic BP, mmHg	91 ± 13	90 ± 16	0.8
BMI, Kg/m^2^	26.4 ± 2.6	25 ± 3.4	0.3
Waist circumference, cm	94 ± 9	94 ± 9	0.7
HbA1c, %	5.4 ± 0.5	5.7 ± 0.6	0.8
Diabetes, %	0	0	1.0
Hypertension, %	60	60	1.0
Sedentary lifestyle, %	60	50	0.5
Smoking habit, %	50	60	0.8
Killip I,%	90	90	1.0
MI mass by cardiac CMRi, g	10.3 (13)	11.9 (12)	0.6
Reperfusion therapy, %	82	85	0.6
GFR (MDRD), mL/min	80 ± 17	78 ± 15	0.6
Glycaemia at admission, mg/dL	119 ± 20	125 ± 21	0.5
LDL-C, mg/dL	121 ± 35	129 ± 27	0.7
HDL-C, mg/dL	34 ± 6	34 ± 6	0.9
Triglycerides, mg/dL	122 ± 88	148 ± 60	0.3
Hs-CRP, g/L	0.7 ± 0.6	0.7 ± 0.5	0.9
ISi at admission, 10^−4^.kg^−1^.min^−1^/(μU/mL)	2.8 ± 1.8	2.8 ± 1.3	1.0
ISi at the 5^th^ day, 10^−4^.kg^−1^.min^−1^/(μU/mL)	3.1 ± 1.9	2.5 ± 0.9	0.030
Delta ISi, %	20 ± 60	−6 ± 28	0.025

S: simvastatin; BP: blood pressure; BMI: body mass index; HbA1c: glycosylated hemoglobin; MI: myocardial infarction; CMRi: cardiac magnetic resonance imaging; PCI: percutaneous coronary intervention; GFR: glomerular filtration rate (by MDRD method); LDL-C: low-density lipoprotein cholesterol; HDL-C: high-density lipoprotein cholesterol; CRP: C-reactive protein; ISi: insulin sensitivity index.

#### Euglycemic hyperinsulinemic clamps

At D2, ISi was not different between patients treated with simvastatin 10 or 80 mg (Supplementary Table 1). Between D2 and D5, the group treated with 10 mg increased ISi from 2.8 ± 1.8 to 3.1 ± 1.9 10^−4^.kg^−1^.min^−1^/(μU/mL), and patients who took 80 mg decrease from 2.8 ± 1.3 to 2.5 ± 0.9 10^−4^.kg^−1^.min^−1^/(μU/mL) (p < 0.05 for both). The differences in Delta ISi between simvastatin groups were confirmed by both Student’s t-test (p = 0.04) and ANCOVA adjusted for sex, age, and ISi at D2 (p = 0.025).

### Observational substudy

#### Baseline characteristics

As commented above, patients were grouped according to the dose of simvastatin during hospitalization. There was no significant difference across groups (both in the whole cohort and in non-diabetics) regarding clinical characteristics as well as infarction mass estimated by MRi, reperfusion therapy, and in-hospital use of beta-blockers, clopidogrel, and AAS or tirofiban (Table 2). Of note, time to reperfusion therapy after the onset of chest pain and medications after discharge were also similar across groups. No statin-associated muscle symptoms were observed in simvastatin users, even in those at 80 mg/day.

**Table 2:** Baseline characteristics of non-diabetic STEMI patients enrolled into the observational substudy.

	Control	S 20 mg	S 40 mg	S 80 mg	*p*-value
Total number of non-diabetic participants	131	77	117	50	
Age, years	63 ± 12	64 ± 12	61 ± 11	63 ± 13	0.5
Male, %	81	71	77	85	0.2
BMI, Kg/m^2^	26 ± 4	27 ± 4	26 ± 5	26 ± 5	0.4
Waist circumference, cm	95 ± 10	95 ± 10	95 ± 11	97 ± 12	0.6
HbA1c, %	5.7 ± 0.4	5.8 ± 0.4	5.5 ± 0.6	5.6 ± 0.6	0.4
Diabetes, %	0	0	0	0	1.0
Prior MI, %	5	8	6	7	0.2
Smoking habit, %	42	46	40	37	0.4
Hypertension, %	46	49	42	43	0.4
Sedentary lifestyle, %	52	45	61	60	0.1
Metabolic syndrome, %	17	18	20	19	0.6
Systolic BP, mmHg	134 ± 30	136 ± 28	140 ± 29	131 ± 29	0.6
Diastolic BP, mmHg	83 ± 20	85 ± 15	88 ± 19	82 ± 18	0.6
Heart rate, bpm	74 ± 18	76 ± 15	75 ± 16	76 ± 16	0.8
Pain-reperfusion time, min	193 ± 178	190 ± 196	194 ± 135	185 ± 132	0.3
Killip I,%	91	88	89	92	0.7
Peak of CK-MB, ng/mL	273 ± 213	279 ± 214	259 ± 169	258 ± 179	0.2
Anterior wall MI, %	55	56	49	50	0.5
Primary PCI, %	24	23	26	22	0.6
Tenecteplase, %	64	65	69	60	0.3
**In-hospital medications**					
AAS, %	97	96	100	100	0.3
Clopidogrel, %	86	78	86	90	0.2
Tirofiban, %	5	6	6	6	0.9
Beta-blockers, %	71	75	69	67	0.4
ARBs or ACEi, %	65	60	62	60	0.8
**Discharge medications**					
ARBs or ACEi, %	95	92	90	93	0.8
Beta-blockers, %	83	79	85	84	0.6
Ca^2+^ channel blockers, %	17	19	21	19	0.9
Nitrates, %	21	21	16	19	0.5
Simvastatin, %	98	100	99	100	0.9

Characteristics of the non-diabetic participants. S: simvastatin; BMI: body mass index; HbA1c: glycosylated hemoglobin; MI: myocardial infarction; BP: blood pressure; PCI: percutaneous coronary intervention; ARBs: angiotensin receptor blockers; ACEi: angiotensin converting enzyme inhibitors.

#### Glucose-insulin homeostasis

At D1, there were no differences between groups in terms of plasma glycaemia, insulin, or C-peptide levels of non-diabetic patients. HOMA2S and HOMA2B were also not different (Table 3). However, between D1 and D5, the controls and the S80 groups presented opposing behaviors on glucose metabolism, followed by an intermediate profile in the S20 and S40 groups. While in the control group glycaemia decreased by 15 ± 46 mg/dL, HOMA2S increased by 40 ± 145% and HOMA2B decreased by 38 ± 100%; in the S80 group glycaemia increased by 1 ± 33% (p = 0.01), HOMA2S decreased by 2 ± 89% (p = 0.001) and HOMA2B increased by 12 ± 100% (p = 0.001).

**Table 3:** Markers of insulin sensitivity and secretion across treatment groups in non-diabetics.

	Control	S 20 mg	S 40 mg	S 80 mg	p-value
Glycaemia D1, mg/dL	125 ± 40	118 ± 32	127 ± 30	120 ± 26	0.5
Insulin D1, µI/mL	24 ± 25	28 ± 32	26 ± 25	25 ± 28	0.6
C-peptide D1, ng/dL	5 ± 3	6 ± 4	5 ± 3	5 ± 3	0.7
HOMA2S D1, %	65 ± 60	66 ± 70	58 ± 49	72 ± 70	0.8
HOMA2B D1, %	117 ± 77	118 ± 90	115 ± 84	118 ± 108	0.8
Disposition index D1, %	41(34)	38(43)	34(38)	31(29)	0.23
Delta Glycaemia D1–D5, mg/dL	−15 ± 46	−12 ± 31	−5 ± 35	1 ± 33	0.01
Delta HOMA2S D1–D5, %	40 ± 145	22 ± 117	16 ± 61	−2 ± 89	0.001
Delta HOMA2B D1–D5, %	−38 ± 100	−23 ± 91	−4 ± 82	12 ± 100	0.001
Disposition index D1–D5, %	17(34)	17(41)	16(31)	3(34)	0.007

S: simvastatin; D1: first day (admission) after STEMI; D5: fifth day after STEMI; HOMA2S: Homeostasis modeling assessment-2 of insulin sensitivity; HOMA2B: Homeostasis modeling assessment-2 of insulin secretion; HOMA2S was based on plasma insulin and HOMA2B on plasma C-peptide. Insulin. Disposition index = HOMA2B * HOMA2S/100.

Subsequently, we evaluated whether simvastatin dose could independently predict a reduction in HOMA2S between D1 and D5 in non-diabetics (Supplementary Table 1). Indeed, HOMA2B at D1 (p = 0.0001), the dose of simvastatin (p = 0.009) and the number of components of metabolic syndrome (p = 0.009) were the only independent markers for a fall in HOMA2S between D1 and D5. Particularly, each 20 mg of simvastatin led to an 18% increase in chance of reducing insulin sensitivity from D1 to D5.

**Figure 2:**
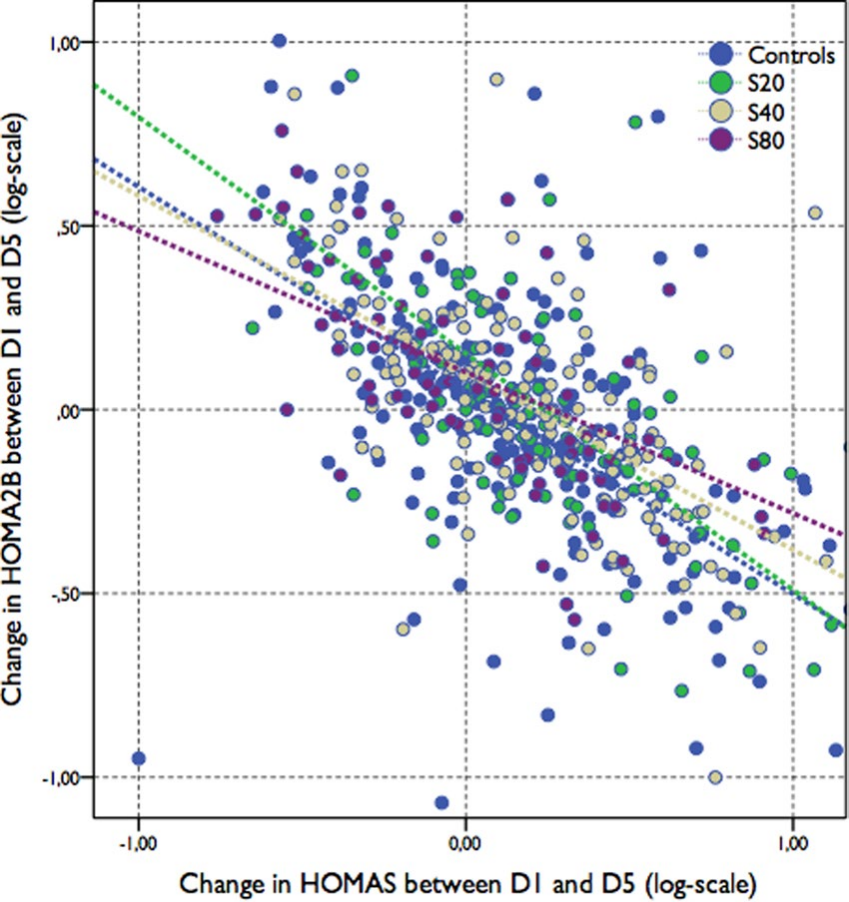
Compensatory changes between HOMA2S and HOMA2B during the first 5 days after MI. A significantly lower slope was found in patients taking simvastatin at 80 mg/day (S80) (β of −33.6 ± 6, p = 0.018) as compared with their counterparts (β of −23.3 ± 10, −18.6 ± 4, and −27.0 ± 6, respectively, in WS, S20 and S40 groups).

It is well known that insulin secretion and sensitivity are connected via a negative feedback loop, where pancreatic β-cells compensate for changes in whole body insulin sensitivity by a proportional and reciprocal change in insulin secretion in a rectangular hyperbolic function (y = constant/x)^20^. Based on this assumption, the product of HOMA2B and HOMA2S, *i*.*e*. the disposition index (DI), remains approximately constant if only one of these parameters is changed. Therefore, in Fig. 2 the expected slope should follow a −45° line. As shown in Fig. 1, we found the following linear regression equations for each group −0.574x + 0.11 (WS), −0.620x + 0.28 (S20), −0.534x + 0.26 (S40) and −0.436x + 0.30 (S80), indicating a dose-dependent change in the coupling between HOMA2S and HOMA2B (p < 0.05). Accordingly, as seen in Table 3, the S80 group showed a significantly impaired change in the DI between D1 and D5 (p = 0.007) when compared to other groups. In addition, by performing PCA, we found two components that summarize roughly 75% of variation of Delta HOMA2S, Delta HOMA2B, and Delta DI (Kaiser-Meyer-Olkin measure of sampling adequacy of 0.51) (Supplementary Fig. 1). While component 1 mostly represent positive values for Delta HOMA2S and Delta DI, component 2 mostly summarizes the positive variance of Delta HOMA2B and Delta DI (Supplementary Table 2). ANCOVA analysis showed that the S80 group presented a significantly lower mean ± SD on component 1 (−0.38 ± 0.2, p = 0.0229) compared to controls (+0.12 ± 0.3), S20 (+0.13 ± 0.3) and S40 (+0.01 ± 0.2). On component 2, however, the groups were equilibrated. Together, these findings suggest that, compared to the controls, S20 and S40 groups, the major source of variance of S80 group is related to HOMA2S impairment.

## Discussion

Although it is increasingly more evident that statins may favor the incidence of diabetes after prolonged treatment^11^, it was still unclear whether statins could derange glucose homeostasis after short-term initiation of therapy. In a small trial with atorvastatin, after two months of treatment, there was reduced insulin sensitivity and C-peptide as well as increased blood glucose and HbA1c in a dose-dependent manner^13^. In another study, 156 patients with MI received atorvastatin 20 or 40 mg/day for one year. Patients with the higher statin dose had a greater increase in plasma glucose, insulin, C-peptide levels and progression of insulin resistance as estimated by the quantitative insulin sensitivity check index (QUICKI) score^21^.

As commented above, cellular model data indicate that statins can readily reduce the phosphorylation of insulin receptor tyrosine, the activation of MAP kinase in fibroblasts and inhibit glucose-induced calcium [Ca^2+^] signaling^14,15^. Through this set of mechanisms, statins would potentially prevent the post-translational prenylation of small G-proteins which is required for membrane targeting and activation of both insulin secretion in pancreatic β-cells and insulin signal transduction in adipocytes^22,23^. To the best of our knowledge, the present study is the first to demonstrate in STEMI patients that a rapid deterioration of insulin signaling and secretion can really be observed soon after statin treatment.

In the very early phase of MI, IS declines because of a spectrum of mechanisms that include the neurohumoral activation and acute phase of inflammatory response^3,24,25^. Although statins can quickly reduce inflammatory activity^26^ and sympathetic activation^27^, its above-commented ability to inhibit prenylation seems to predominate in the modulation of IS during acute stress according to our EHC data^28^. Indeed, in *in vitro* model with 3T3-L1 adipocytes, statins promote downregulation of insulin-sensitive GLUT-4 and up-regulation of GLUT-1; these effects are reversed by mevalonate, demonstrating that inhibition of isoprenoid biosynthesis causes insulin resistance in adipocytes^29^. In our observational substudy, IS estimated by HOMA2S decreased and insulin secretion estimated by HOMA2B increased in a directly proportional manner; this pairing resulted in an unchanging DI. At the higher simvastatin dose (80 mg/day), however, this balance was lost probably reflecting the statin-mediated inhibition of insulin secretion.

EHC remains as the gold standard for evaluating IS even in stress conditions due to the suppression of hepatic glucose secretion by constant infusion of insulin. Yet, EHC is a laborious and time-consuming procedure, unsuitable to be executed in large sample sizes, particularly in STEMI patients. With this in mind, we recently tested the accuracy of surrogate homeostasis models in STEMI patients and found that the HOMA2S can be used with accuracy, thus representing a viable tool for larger clinical studies^16^. Following this strategy, we were able to verify the dose-dependent effect on insulin sensitivity and secretion and to detect in multivariate models that besides statin dose, the presence of metabolic syndrome components and HOMA2B obtained at admission are independent predictors of IS decline after MI.

This study was designed to test the existence of an inverse and dose-dependent association between statin therapy initiated after STEMI and insulin sensitivity. For this, we used two distinct methods for assessing insulin sensitivity and we confirmed the hypothesis. Nevertheless, important limitations need to be recognized and considered when interpreting our findings. First, the study exclusively enrolled STEMI patients to homogenize the sample with individuals with a greater extent of myocardial injury. This strategy prevents our findings from being extrapolated to other acute coronary syndromes. Second, all patients used exclusively simvastatin and there may be heterogeneity between statins in the effect on glucose homeostasis. Third, because of the limited sample size, we cannot verify if a clinical impact exists after the acutely statin-induced IR. This hypothesis, needs to be properly tested and our study is not fit for it. Finally, although no difference was apparent between patients grouped according to statin therapy in the observational substudy, we cannot exclude selection bias. However, since the findings are equivalent in the randomized controlled substudy and in the observational substudy, altogether this set of data adds consistency for the statin and insulin sensitivity interaction during STEMI.

## Conclusion

In conclusion, statin therapy initiated upon admission of STEMI quickly reduces insulin sensitivity in a dose-dependent manner as estimated by both EHC and HOMA2S% assessments.

### Ethics approval and consent to participate

Institutional Ethics Committee approved protocols of both substudies and all patients signed an informed consent term. The study was registered at ClinicalTrials.gov by the numbers NCT02062554 and NCT01205347.

### Consent for publication

All authors have read and approved the manuscript submission as it is.

### Registration numbers

At ClinicalTrials.gov: NCT01205347, at September 20, 2010 and NCT02062554 retrospectively registered at February 13, 2014.

## Supplementary information

Supplementary results

## Data Availability

The datasets generated and/or analyzed during the current study are not publicly available due to ongoing proprietary work but are available from the corresponding author on reasonable request.
