# Supplementary material for: Statin Short-term Inhibition of Insulin Sensitivity and Secretion During Acute Phase of ST-Elevation Myocardial Infarction
Source: Sci Rep. 2019 Nov 8;9:16401. doi: 10.1038/s41598-019-52111-x (PMC6841947; doi:10.1038/s41598-019-52111-x)

Statin Short-term Inhibition of Insulin Sensitivity and Secretion During Acute Phase of ST-Elevation Myocardial Infarction

Andrei C Sposito[1], Luiz Sergio F Carvalho[1], Filipe A. Moura[2], Alessandra M. Campos-Staffico[1], Riobaldo MR Cintra[1], Wilson Nadruz[1], Osorio R Almeida[3], Jose C Quinaglia e Silva[3]

SUPPLEMENTARY DATA

Supplementary Table 1. Independent predictors of HOMA2S fall between D1 and D5 in non-diabetics.

	**B**	***p-value***	**Exp(B)**	**95% C.I. for Exp(B)**	
				**Lower**	**Upper**
Male gender	-0.29	0.38	0.748	0.391	1.429
Age	-0.012	0.284	0.988	0.966	1.010
HbA1c	-0.291	0.235	0.747	0.462	1.208
HOMA2B at D1	0.025	<0.001	1.026	1.019	1.032
Metabolic syndrome (number of components)	0.366	0.009	1.442	1.094	1.901
Simvastatin dose	0.009	0.009	1.009	1.001	1.018
Constant	-0.703	0.631	0.495		

D1: first day (admission) after STEMI; HbA1c: glycosylated hemoglobin; HOMA2B: Homeostasis modeling assessment-2 of insulin secretion.

Supplementary Table 2. Linear correlation coefficients between the two components derived after Principal Component Analysis and the change in HOMA2S, HOMA2B and Disposition Index between D1 and D5.

	**Component 1**	**Component 2**
Delta HOMA2S	0.7210	-0.0397
Delta HOMA2B	-0.3443	0.8458
Delta Disposition Index	0.6014	0.5320

Supplementary Figure 1. Distribution of coefficients of two components derived after Principal Component Analysis and the change in HOMA2S, HOMA2B and Disposition Index between D1 and D5.

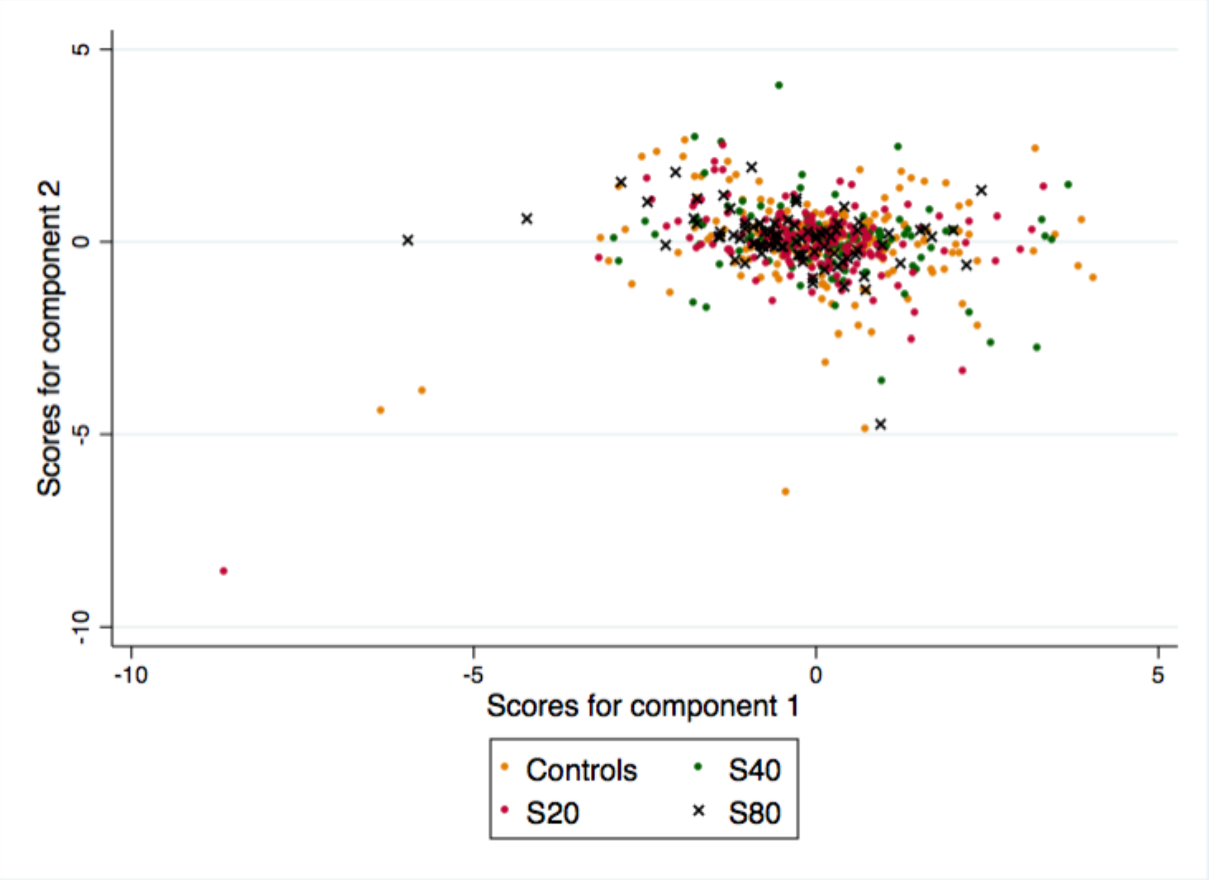

Supplement: Supplementary file 1 — Supplementary results [file 41598_2019_52111_MOESM1_ESM.pdf]
